# N–3 Fatty Acid Supplementation in Mothers and Infants for Childhood Psychomotor and Cognitive Development: An Updated Systematic Review and Meta‐Analysis

**DOI:** 10.1111/mcn.13767

**Published:** 2024-11-28

**Authors:** Yingyu Liu, Lijun Zhong, Zhouyang Sun, Yuan Feng, Qianlu Ding, Yujian Zhang

**Affiliations:** ^1^ Division of Surveillance and Evaluation Chinese Center for Health Education Beijing China; ^2^ Department of Epidemiology and Biostatistics School of Public Health Jilin University Changchun China; ^3^ Department of Cancer Prevention and Control, Sichuan Clinical Research Center for Cancer Sichuan Cancer Hospital & Institute, Sichuan Cancer Center, Affiliated Cancer Hospital of University of Electronic Science and Technology of China Chengdu China

**Keywords:** cognition, fatty acids unsaturated, growth and development, infant formula, intelligence, meta‐analysis, Wechsler scales

## Abstract

Long‐chain n–3 polyunsaturated fatty acid (PUFA) consumption in maternal and infants has been positively associated with cognitive and visual development. Tails even meta‐analysis showed mixed results. To evaluate the effects of maternal and infant n–3 PUFA supplementation on childhood psychomotor and cognitive development, PubMed, Embase, the Cochrane Library, PsycINFO and clinicaltrials.gov were searched. Randomized controlled trials were included to evaluate the effect on child cognitive and psychomotor outcomes of n–3 PUFA supplementation in mothers or infants (age ≤ 2 years). Findings were pooled with mean differences (MD) with 95% confidence intervals (95% CIs). Heterogeneity was explored using *I* ^2^ and subgroup analyses, stratified for maternal (pregnancy and/or lactation) and infant (preterm infant and term infant). We identified 47 articles, with 14 trials on mothers and 33 on infants. Pooled results showed that infants' mental development index (MDI) increased with n–3 PUFA supplementation (MD = 2.91, 95% CI: 1.32–4.51, *I* ^2^ = 65.1%). Subgroup analysis of MDI also demonstrated a benefit in preterm infants (MD = 4.16, 95% CI: 1.40–6.93, *I* ^2^ = 49.5%) and term infants (MD = 2.28, 95% CI: 0.27–4.29, *I* ^2^ = 70.1%). No significant association was found in subgroup analyses of supplementation to mothers during pregnancy or lactation period. Supplementation did not increase the psychomotor development index (PDI) in the mother or infant group. Language composite score increased for infants whose mothers accepted supplementation in pregnancy or breastfeeding (MD = 8.57, 95% CI: 5.09–12.04, *I* ^2^ = 70.2%). The cognitive composite score did not improve in any subgroup. Intelligence Quotient (IQ) increased in the infants' group with n–3 PUFA supplementation (MD = 2.54, 95% CI: 0.45–4.63, *I* ^2^ = 66.0%). Furthermore, IQ in term infants also improved (MD = 2.91, 95% CI: 0.24–5.57, *I* ^2^ = 69.2%). The funnel plot and Egger's test confirmed no publication bias in any endpoints. Supplementation with n–3 PUFA during pregnancy or breastfeeding in mothers has increased language abilities. Furthermore, direct supplementation in term infants can improve intelligence in later childhood. However, insufficient evidence supports the claim that supplementation improves cognitive abilities.

## Summary

- Long‐chain n–3 polyunsaturated fatty acid (PUFA) is a significant building structure of membrane phospholipids of the brain and necessary for neuronal function.
- Tails even meta‐analysis about PUFA consumption in maternal and infants cognitive and visual development showed mixed results.
- Our findings showed that supplementation with n–3 PUFA during pregnancy or breastfeeding in mothers has increased language abilities and supplementation in term infants can improve intelligence in later childhood.
- Insufficient evidence supports the claim that supplementation improves cognitive abilities.

## Graphical abstract

This systematic review and meta‐analysis showed that supplementation with n–3 polyunsaturated fatty acid during pregnancy or breastfeeding in mothers has increased language abilities. Furthermore, direct supplementation in term infants can improve intelligence in later childhood. However, insufficient evidence supports the claim that supplementation improves cognitive abilities.

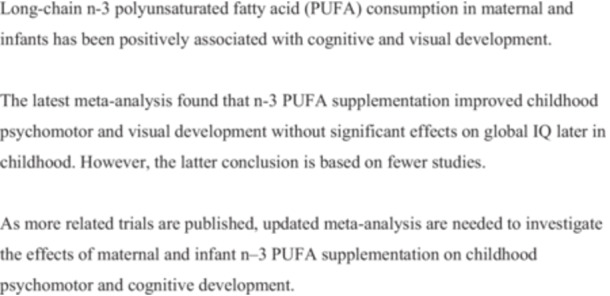

## 1 Introduction

Long‐chain n–3 (ω‐3) polyunsaturated fatty acid (PUFA), particularly docosahexaenoic acid (DHA), a significant building structure of membrane phospholipids of the brain and necessary for neuronal function, is essential for the development and function of the brain and retina. It is known to be involved in neurotransmission, neurogenesis, signal transduction, gene expression, and anti‐inflammation (Chalon 2006; Innis 2007; Lauritzen, 2001; Simopoulos 2002). DHA is a non‐essential fatty acid that can be synthesized from α‐linolenic acid, but conversion efficiency remains notably low (Bourre 2006). Therefore, it is crucial to ensure an adequate intake of DHA through dietary sources rich in this fatty acid, such as fatty fish or DHA supplements.

Pregnancy and infancy are crucial periods for the formation of the brain that lay the foundation for developing motor, cognitive, and socio‐emotional skills throughout childhood and adulthood (Garey 1984; Martinez 1992). DHA concentrations in brain tissue increased linearly from late pregnancy to 2 years of age (Martinez 1992), which transferred across the placenta to the foetus in high amounts during pregnancy or obtained from breast milk or enriched formula for infants (Haggarty et al. 1997; Khandelwal et al. 2023). Consensus recommendations and practice guidelines for healthcare providers supported by the World Association of Perinatal Medicine, the Early Nutrition Academy, the Child Health Foundation, and the Food and Agriculture Organization of the United Nations (FAO) suggested the foetus and neonate should receive LC‐PUFA in amounts sufficient to support optimal visual and cognitive development. Moreover, pregnant and lactating women should aim to achieve a daily intake of DHA (Koletzko et al. 2008; Ronald and Kleinman 2008).

However, supplementation with DHA alone has been found to be associated with necrotizing enterocolitis in preterm infants (Alshaikh et al. 2023).

Based on the epidemiologic basis above, many trials focused on maternal or infant n–3 PUFA supplementation and childhood cognitive development but showed mixed results, even meta‐analysis. The latest meta‐analysis of 38 trials found that n–3 PUFA supplementation improved childhood psychomotor and visual development without significant effects on global IQ later in childhood. However, the latter conclusion is based on fewer studies (Shulkin et al. 2018). After that, a meta‐analysis of 11 trials showed no significant association between DHA/EPA supplementation in pregnant or breastfeeding women and the cognitive performance of children (Lehner et al. 2021).

To include more recently published trials, we updated the meta‐analysis to investigate the effects of maternal and infant n–3 PUFA supplementation on childhood psychomotor and cognitive development.

## 2 Methods

### 2.1 Study Registration

We followed the Cochrane Handbook of Systematic Reviews of Interventions (Higgins and Green 2008) and the Preferred Reporting Items for Systematic Reviews and Meta‐Analyses (PRISMA) guidelines (Moher, Liberati, and Fau 2009) during this meta‐analysis. The protocol of this systematic review and meta‐analysis was registered on the INPLASY website (https://inplasy.com/inplasy-2024-5-0131/) and INPLASY registration number is INPLASY202450131.

### 2.2 Search Strategies

Based on search strategies of previous meta‐analysis (Shulkin et al. 2018), we performed supplemented electronic searches of PubMed, Embase, the Cochrane Library, PsycINFO, and clinicaltrials.gov from January 1, 2016 through January 24, 2024.

To avoid missing any trials, we included several common language terms in addition to Medical Subject Heading terms. The main search terms related to following searched strategy: ([omega‐3 fatty acids] or [PUFAs] or [DHA] or [eicosapentaenoic acid] or [fish] or [seafood]) and ([neurodevelopment] or [cognition] or [development] or [vision]) and ([child] or [infant] or [pregnancy] or [lactation]). The study was confined to English publications focusing on humans. The detailed search strategies that we used in each database are presented in Supplement‐PART 1. Moreover, reference lists from reviews, meta‐analyses, and the retrieved articles were searched to identify further relevant studies.

### 2.3 Study Inclusion and Exclusion Criteria

To be eligible for inclusion, the studies had to meet the following criteria based on the PICOS principle:

1. *Participants (P):* Pregnant mothers, nursing mothers, or children aged < 2 years, generally healthy subjects.
2. *Intervention (I) and Comparator (C):* With or without supplementation or fortification with n–3 PUFA (DHA, EPA, or a combination, or with other n–3 or n–6 PUFAs) for a minimum duration of 3 months.
3. *Outcome (O):* Assessment of cognitive development using quantitative and standardized measures. The relevant infant development measures included Bayley Scales of Infant Development (BSID‐II [San Antonio 1993] or BSID‐III [San Antonio and Bayley 2006]), Intelligence quotient (IQ) based on the Wechsler Intelligence Scale for Children (WISC) (Wechsler 2003), the Wechsler Preschool and Primary Scale of Intelligence (WPPSI) (Wechsler 2012), and the Wechsler Abbreviated Scale of Intelligence (WASI) (Wechsler 1999).
4. *Study design (S):* Randomized controlled trials.

If there were several published reports stemming from the same study, only the one containing the most comprehensive details or original data and outcomes was considered for inclusion.

Studies were excluded if (1) subjects with a specifically defined health problem or received supplementation as a treatment or secondary prevention; (2) trials with supplements that contained additional active ingredients in addition to n–3 PUFA only and were given to the intervention group, included trials that compared breastfed to formula‐fed infants; (3) not offer essential or clear information of reported findings to determine differences in group mean values at follow‐up.

### 2.4 Study Selection, Data Extraction, and Quality Assessment

Based on the specified inclusion and exclusion criteria listed above, one investigator (L.Y.Y.) screened the titles and abstracts of all retrieved articles. And two investigators (L.Y.Y. and Z.Y.J.) independently reviewed the full text of potentially eligible articles and performed the data extraction and quality assessment for studies finally included, any differences were resolved by consensus.

A pro‐form‐designed for data extraction was as followings: (1) bibliographic details: first author, publication year, trial number, study sites, study period; (2) trials design: inclusion and exclusion criteria, similarity of baseline characteristics, sample size, follow‐up time; (3) details of intervention and control group: main intervention, the type and daily doses of supplementation, supplementation period, supplementation duration, type of control, compliance, drop‐out; (4) outcomes: type of test, age at assessment, mean and statistical values; and (5) quality assessment: according to the Cochrane Risk of Bias tool (Higgins and Green 2011), assessed from following items: generation of a randomization sequence, allocation concealment, blinding, incomplete outcome data, selective reporting, and other bias.

### 2.5 Statistical Analyses

Stata (version 15.0) software was used in statistical analyses and meta‐analysis. The mean differences (MD) with 95% confidence intervals (95% CIs) at follow‐up between the intervention and control groups were utilized for individual and pooled statistics. For means, missing information can be obtained by estimating from figures using WebPlotDigitizer 4.7. If necessary, standard errors were transformed into standard deviations (SD). Median values (and ranges) were converted to mean and SD, or the SD was estimated if not reported for continuous variables.

Statistical heterogeneity was assessed with the *I* ^2^ statistic. If a significant statistical heterogeneity was present (*I* ^2^ > 50%), the outcomes were combined using the random‐effect model assuming that studies were drawn from unequal populations. Otherwise, the fixed‐effect model was used.

Heterogeneity was also investigated by using subgroup analyses in both maternal and infant groups across supplementation periods. For maternal, subgroup analyses were grouped according to period of supplementation (pregnancy and/or lactation). And for infant, we detected separately the effects of n–3 PUFA supplementation for preterm infant and term infant.

We conducted a planned analysis to examine the effect of n–3 PUFA treatment on the cognitive and IQ development. For the measured scale for cognitive development, because the different versions of the BSID involve different procedures for administration and scoring, the data generated from BSID‐II or BSID‐III were considered as separate subgroups in the meta‐analysis. For studies based on BSID‐II, we analysed the outcome on the mental development index (MDI) and the psychomotor development index (PDI). And for studies based on BSID‐III, we further evaluated outcomes of cognitive, language, and motor abilities and used the BSID‐III motor facet as the PDI to estimate and pool. For the IQ development, we also conducted subgroup analysis by different measurement scales.

Data about the primary and secondary outcomes in all of the n–3 PUFA groups were included if multiple dose levels or multiple types of n–3 PUFA were considered as the intervention. Whenever clinical trials referred to the same populations at different follow‐up periods, we used only the population with the longest follow‐up period to avoid data duplication.

The risk of publication bias was assessed by visualization of funnel plots, followed by Egger's test. The significance level was set at *p* < 0.05.

## 3 Results

### 3.1 Study Characteristics

**Figure 1:**
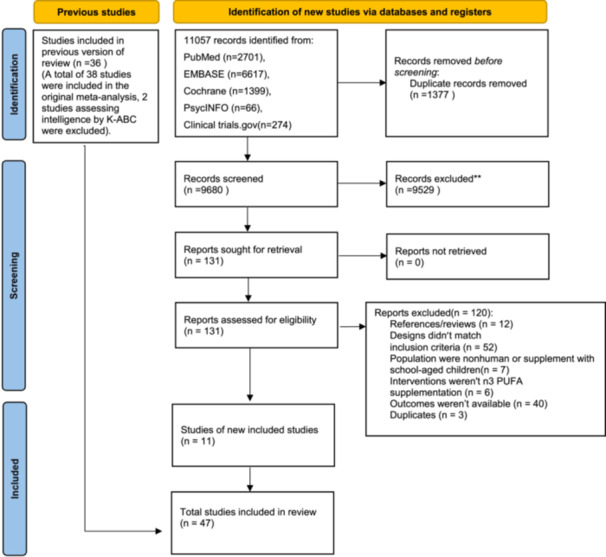
PRISMA flow diagram of search strategy and identified studies.

From 11,057 unique articles, we identified 11 eligible trials and 36 trials included in previous meta‐analyses (Shulkin et al. 2018) for inclusion and pooled them together with a total of 47 (Figure 1). These studies included 8187 unique participants, with 14 trials on mothers, 21 on term infants, and 12 on preterm infants (Table 1 and Supporting Information S1: Tables S1–S3). Most trials were performed in developed countries with high incomes, although trials were also included in Bangladesh, Taiwan, and Mexico. Most Western trials' population was predominantly white, and only five trials evaluated black participants. In 17 of the 47 trials, maternal education was rated as high degree (mean ≥ 14 years of education or > 60% had a college degree or above), nine evaluated mothers with average education (mean 12–13.9 years of education), five evaluated mothers with low education (not completing high school, mean < 12 years of education), and 16 trials were missing sufficient data to evaluated education level of mothers. Among maternal supplementation trials, the median (min–max) baseline maternal age was 30.1 (22.7–33.2) years and supplementation duration was 20 (12–36) weeks. Among term‐infant supplementation trials, most started supplementation from birth (*n* = 14), four started within 6 weeks of breastfeeding, and three started after 4–6 months of breastfeeding, and supplementation duration was 46 (13.6–52) weeks. Among preterm infant supplementation trials, the median age was 29.9 (28.6–35.6) weeks gestation at birth, and the duration of supplementation was 35.7 (9–61.9) weeks. Across all trials, participants in control groups were provided either vegetable/corn/olive/soy oil or standard formula/fortified food without DHA/EPA. In addition, compared with the previous meta‐analysis (Shulkin et al. 2018), there were no new studies on the assessment of visual acuity, relevant results are presented in the footnotes of Supporting Information S1: Tables S1–S3.

**Table 1:** Summary of characteristics of 47 randomized controlled trials testing effects of long‐chain n–3 PUFA supplementation on childhood cognitive and visual development.

Characteristics	Maternal (*n* = 14)	Term infant (*n* = 21)	Preterm infant (*n* = 12)
Location	Australia (*n* = 3), Bangladesh (*n* = 1), Mexico (*n* = 1), USA/Canada (*n* = 4), Western Europe (*n* = 5)	Australia (*n* = 4), China (*n* = 1), USA/Canada (*n* = 12), Western Europe (*n* = 4)	Australia (*n* = 1), Mixed (*n* = 4), Taiwan (*n* = 1), USA (*n* = 3), Western Europe (*n* = 3)
Total participants, *n*	3544	2461	2182
Participants per trial, *n* Median (min–max)	140 (30–730)	92 (19–309)	99 (27–480)
Baseline age, median (min–max)	30.1 (22.7–33.2) years	From birth (*n* = 14), after ≤ 6 weeks of breastfeeding (*n* = 4), after 4–6 months of breastfeeding (n = 3)	29.9 (28.6–35.6) weeks gestation at birth
Race/ethnicity	Asian (*n* = 1), black (*n* = 2), Hispanic (*n* = 1), white (*n* = 10)	Asian (*n* = 1), black (*n* = 2), white (*n* = 17), missing (*n* = 1)	Asian (*n* = 1), black (*n* = 1), white (*n* = 9), missing (*n* = 1)
Maternal education level	High (*n* = 6), average (*n* = 5), low (*n* = 1), missing (*n* = 2)	High (*n* = 8), average (*n* = 3), low (*n* = 3), missing (*n* = 7)	High (*n* = 3), average (*n* = 1), low (*n* = 1), missing (*n* = 7)
Supplementation duration, week, median (min–max)	20 (12–36) weeks	46 (13.6–52) weeks	35.7 (9–61.9) weeks
Type of control	Vegetable oil (*n* = 10), fortified food without DHA/EPA (*n* = 4)	Formula (*n* = 12), olive oil (*n* = 2), fortified food without DHA/EPA (*n* = 7)	Formula (*n* = 7), fortified food without DHA/EPA (*n* = 5)
Outcomes assessed	BSID (*n* = 9), TAC (*n* = 2), VEP (*n* = 4), WISC/WASI/WPPSI (*n* = 2)	BSID (*n* = 11), HOTV19 (*n* = 1), Stanford‐Binet (*n* = 1), TAC (*n* = 3), VEP (*n* = 8), WISC/WASI/WPPSI (*n* = 4)	BSID (*n* = 8), TAC (*n* = 2), VEP (*n* = 3), WISC/WASI/WPPSI (*n* = 3)
Latest age at outcome assessment, months, median (min–max)	BSID: 18 (10–30) IQ: 108 (72–144), visual acuity: 4 (2–64)	BSID: 18 (6–24) IQ: 71 (48–108), visual acuity: 12 (4–48)	BSID: 18 (12–24) IQ: 96 (60–130), visual acuity: 6 (4–24)

### 3.2 The Methodological Quality of Included Studies

**Figure 2:**
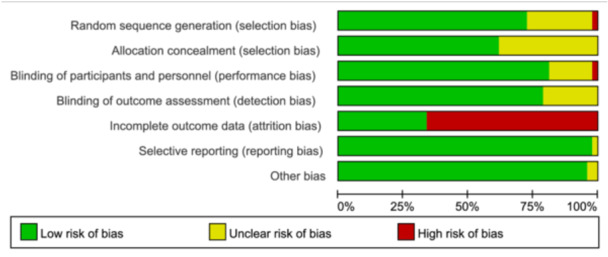
Risk of bias summary of included RCTs.

Among the included 47 RCTs, 34 trials reported the details of randomization, 29 trials used allocation concealment, 38 studies clearly provided double blinding to their participants and personnel, 37 studies were blinding for outcome assessment, Selective reporting and other bias were not found in 46 studies and 45 studies, respectively. Relatively high loss to follow‐up was seen in 31 trials, although there was generally similar drop‐out rates between n–3 PUFA supplementation and placebo groups (Figure 2 and Supporting Information S1: Figure S1).

### 3.3 Effects of n–3 PUFA Supplementation on MDI‐Based on BSID‐II

**Figure 3:**
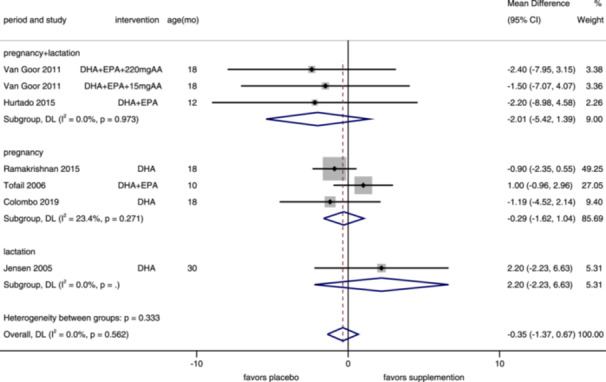
Forest plot of mental development index for maternal supplementation. Effects of n–3 PUFA supplementation on MDI (weighted mean difference) in randomized controlled trials. These analyses included seven intervention arms from six studies, with an overall pooled result across all supplementation periods of −0.35 (95% CI: −1.37, 0.67). Findings were pooled using random effects meta‐analysis. Shaded squares represent the weight of each study, and dotted vertical lines and diamonds represent the pooled central estimate and its 95% CI, respectively, for each group. Age(mo)‐age at outcome assessment, months. *Note:* Weights and between‐subgroup heterogeneity test are from random‐effects model.

**Figure 4:**
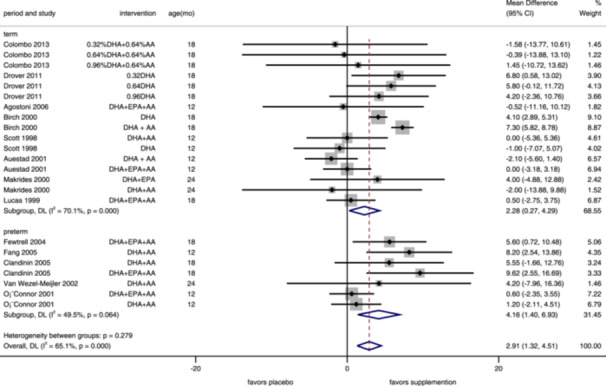
Forest plot of mental development index for infants' supplementation. Effects of n‐3 PUFA supplementation on MDI (weighted mean difference) in randomized controlled trials. These analyses included 23 intervention arms from 13 studies, with an overall pooled result across all supplementation periods of 2.91 (95% CI: 1.32–4.51). Findings were pooled using random effects meta‐analysis. Shaded squares represent the weight of each study, and dotted vertical lines and diamonds represent the pooled central estimate and its 95% CI, respectively, for each group. Age(mo)‐age at outcome assessment, months. *Note:* Weights and between‐subgroup heterogeneity test are from random‐effects model.

Thirty intervention arms reported MDI based on BSID‐II, and overall pooled MDI was increased in the supplementation of the n–3 PUFA group (MD = 1.91, 95% CI: 0.48–3.34, *I* ^2^ = 74.0%) (Supporting Information S1: Figure S2A). We conducted an updated pooled analysis on MDI of maternal supplementation by period (Figure 3). Six trials, including seven intervention arms, reported MDI, assessed in children aged 10–30 months (median 18). Overall (MD = −0.35, 95% CI: −1.37, 0.67, *I* ^2^ = 0%) and subgroup results (pregnancy and lactation: MD = −2.01, 95% CI: −5.42, 1.39, *I* ^2^ = 0%; pregnancy: MD = −0.29, 95% CI: −1.62, 1.04, *I* ^2^ = 23.4%; lactation: only included one study) were not significantly different between children whose mothers were with or without n–3 PUFA supplementation. And for infants, we found that n–3 PUFA supplementation increased their MDI (MD = 2.91, 95% CI: 1.32–4.51, *I* ^2^ = 65.1%), and the same in the subgroup analysis of studies on both term (MD = 2.28, 95% CI: 0.27–4.29, *I* ^2^ = 70.1%) and preterm (MD = 4.16, 95% CI: 1.40–6.93, *I* ^2^ = 49.5%) infants (Figure 4). According to the funnel plot (Supporting Information S1: Figure S3) and Egger's test (*p* = 0.389), there was no significant publication bias between included trials.

### 3.4 Effects of n–3 PUFA Supplementation on PDI (Based Both on BSID‐II and BSID‐III)

**Figure 5:**
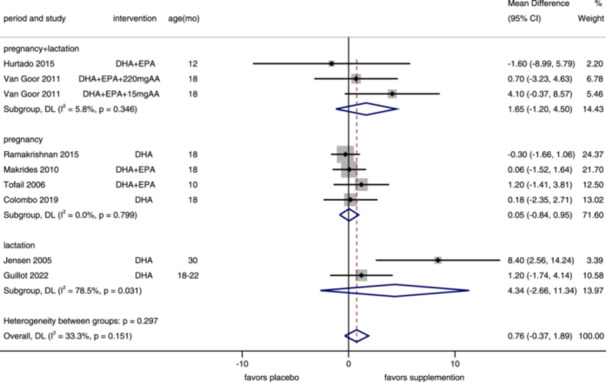
Forest plot of psychomotor development index for maternal supplementation. Effects of n–3 PUFA supplementation on PDI (weighted mean difference) in randomized controlled trials. These analyses included nine intervention arms from eight studies, with an overall pooled result across all supplementation periods of 0.76 (95% CI: −0.37, 1.89). Findings were pooled using random effects meta‐analysis. Shaded squares represent the weight of each study, and dotted vertical lines and diamonds represent the pooled central estimate and its 95% CI, respectively, for each group. Age(mo)‐age at outcome assessment, months. *Note:* Weights and between‐subgroup heterogeneity test are from random‐effects model.

**Figure 6:**
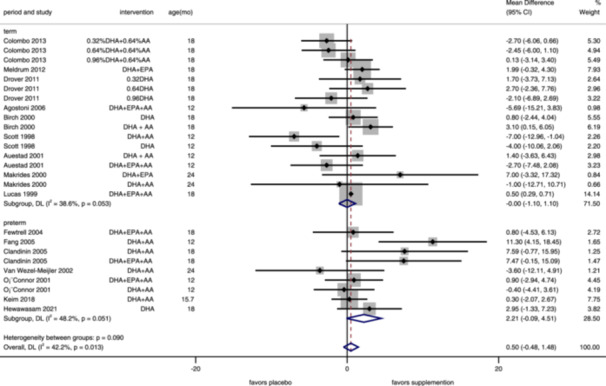
Forest plot of psychomotor development index for infant's supplementation. Effects of n–3 PUFA supplementation on PDI (weighted mean difference) in randomized controlled trials. These analyses included 26 intervention arms from 16 studies, with an overall pooled result across all supplementation periods of 0.50 (95% CI: −0.48, 1.48). Findings were pooled using random effects meta‐analysis. Shaded squares represent the weight of each study, and dotted vertical lines and diamonds represent the pooled central estimate and its 95% CI, respectively, for each group. Age(mo)‐age at outcome assessment, months. *Note:* Weights and between‐subgroup heterogeneity test are from random‐effects model.

Thirty‐five intervention arms reported PDI, including the BSID‐III motor facet (Supporting Information S1: Figure S2B). And assessed in children aged 10–30 months (median 18). No significant difference of PDI in the supplementation of n–3 PUFA group (MD = 0.61, 95% CI: –0.09, 1.31, *I* ^2^ = 38.5%), either maternal periods (MD = 0.76, 95% CI: –0.37, 1.89, *I* ^2^ = 33.3%, Figure 5) or infants' periods (MD = 0.50, 95% CI: –0.48, 1.48, *I* ^2^ = 42.2%, Figure 6). For maternal supplementation, the pooled results of supplementing only during pregnancy (MD = 0.05, 95% CI: −0.84, 0.95, *I* ^2^ = 0%), supplementing only during lactation (MD = 4.34, 95% CI: −2.66, 11.34, *I* ^2^ = 78.5%), and supplementing during both periods (MD = 1.65, 95% CI: −1.20, 4.50, *I* ^2^ = 5.8%) all have shown no significant improvement in PDI (Figure 5). Supplementation did not significantly improve PDI in both term (MD = −0.0, 95% CI: −1.10, 1.10, *I* ^2^ = 38.6%) and preterm infants (MD = 2.21, 95% CI: −0.09, 4.51, *I* ^2^ = 48.2%) (Figure 6). According to the funnel plot (Supporting Information S1: Figure S3B) and Egger's test (*p* = 0.595), no publication bias was found in including studies on PDI.

### 3.5 Effects of n–3 PUFA Supplementation Based BSID‐III

**Figure 7:**
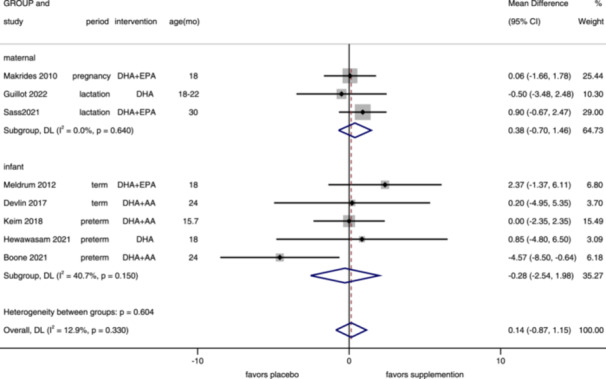
Forest plot of cognitive composite score of BSID‐III. Effects of n–3 PUFA supplementation on cognitive composite score (weighted mean difference) in randomized controlled trials. These analyses included eight intervention arms from eight studies, with an overall pooled result across all supplementation periods of 0.14 (95% CI: –0.87, 1.15). Findings were pooled using random effects meta‐analysis. Shaded squares represent the weight of each study, and dotted vertical lines and diamonds represent the pooled central estimate and its 95% CI, respectively, for each group. Age(mo)‐age at outcome assessment, months. *Note:* Weights and between‐subgroup heterogeneity test are from random‐effects model.

Eight studies reported a cognitive composite‐based third edition of BSID, assessed in children aged 15–30 months (median 19 months). There was no significant improvement in overall (MD = 0.14, 95% CI: −0.87, 1.15, *I* ^2^ = 12.9%), maternal (MD = 0.38, 95% CI: −0.70, 1.46, *I* ^2^ = 0%), and infant (MD = −0.28, 95% CI: −2.54, 1.98, *I* ^2^ = 40.7%) supplementation (Figure 7). Results of all periods showed no significant difference between the n−3 PUFA supplementation group and control group (lactation: MD = 0.60, 95% CI: −0.79, 1.98, *I* ^2^ = 0%; term: MD = 1.62, 95% CI: −1.40, 4.65, *I* ^2^ = 0%; preterm: MD = −1.29, 95% CI: −4.48, 1.90, *I* ^2^ = 53.2%) (Supporting Information S1: Figure S2). The funnel plot (Supporting Information S1: Figure S3) and Egger's test (*p* = 0.525) found no publication bias.

**Figure 8:**
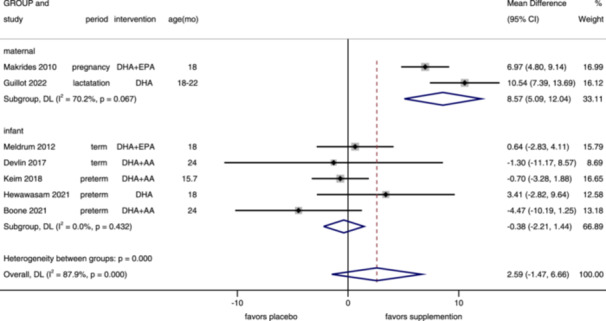
Forest plot of language score of BSID‐III. Effects of n–3 PUFA supplementation on language score (weighted mean difference) in randomized controlled trials. These analyses included seven intervention arms from seven studies, with an overall pooled result across all supplementation periods of 2.59 (95% CI: −1.47, 6.66). Findings were pooled using random effects meta‐analysis. Shaded squares represent the weight of each study, and dotted vertical lines and diamonds represent the pooled central estimate and its 95% CI, respectively, for each group. Age(mo)‐age at outcome assessment, months. *Note:* Weights and between‐subgroup heterogeneity test are from random‐effects model.

Seven studies reported language composite, assessed in children aged 15–24 (median 18 months). No significant difference in overall (MD = 2.59, 95%% CI: −1.47, 6.66, *I* ^2^ = 87.9%). For maternal supplementation period, n–3 PUFA supplementation increased language composite (MD = 8.57, 95% CI: 5.09−12.04, *I* ^2^ = 70.2%), while no significant difference in infants group (MD = −0.38, 95% CI: −2.21, 1.44, *I* ^2^ = 0%), term (MD = 0.43, 95% CI: −2.85, 3.70, *I* ^2^ = 0%) and preterm (MD: −0.73, 95% CI: −4.14, 2.68, *I* ^2^ = 40.1%) (Figure 8 and Supporting Information S1: Figure S2). No publication bias was found by funnel plot (Supporting Information S1: Figure S3D) and Egger's test (*p* = 0.432).

### 3.6 Effects of n–3 PUFA Supplementation on IQ

**Figure 9:**
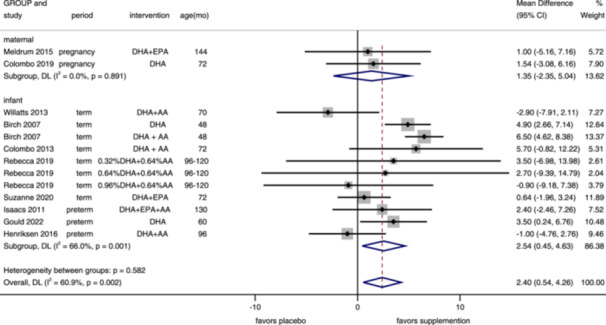
Forest plot of IQ. Effects of n–3 PUFA supplementation on IQ (weighted mean difference) in randomized controlled trials. These analyses included 13 intervention arms from 10 studies, with an overall pooled result across all supplementation periods of 2.40 (95% CI: 0.54–4.26). Findings were pooled using random effects meta‐analysis. Shaded squares represent the weight of each study, and dotted vertical lines and diamonds represent the pooled central estimate and its 95% CI, respectively, for each group. Age(mo)‐age at outcome assessment, months. *Note:* Weights and between‐subgroup heterogeneity test are from random‐effects model.

Ten studies, including 13 intervention arms, reported IQ with WPPSI/WASI/WISC, assessed in children aged 48–144 months (median 72 months). IQ increased in overall (MD = 2.40, 95% CI: 0.54–4.26, *I* ^2^ = 60.9%) and infants' group with n–3 PUFA supplementation (MD = 2.54, 95% CI: 0.45–4.63, *I* ^2^ = 66.0%), while no improvement in maternal group (MD = 1.35, 95% CI: –2.35, 5.04, *I* ^2^ = 0%) (Figure 9). Furthermore, for term infants with n–3 PUFA supplementation, IQ also increased (MD = 2.91, 95% CI: 0.24–5.57, *I* ^2^ = 69.2%), though no influence for supplementation on preterm infants (MD = 1.67, 95% CI: –1.18, 4.52, *I* ^2^ = 38.0%) (Supporting Information S1: Figure S2). No publication bias was found (funnel plot in Supporting Information S1: Figure S3, Egger's test: *p* = 0.091).

## 4 Discussion

This systematic review and meta‐analysis analysed 47 trials during critical periods of brain development, from pregnancy to infancy, and showed some benefits of n–3 PUFA supplementation in later childhood. IQ showed stronger benefits than MDI. No significant association was found for PDI. Supplementation for mothers during pregnancy and lactation showed a significant increase in their children's language composite scores. Supplementation for infants significantly improved their MDI scores and showed a stronger improvement in MDI scores in preterm infants compared with those in term infants. Term infants who received n–3 PUFA supplementation exhibited higher IQ scores in later childhood than infants without additional supplementation. However, no significant effects on neurodevelopmental endpoints or global intelligence except MDI were observed in preterm infants who received n–3 PUFA supplementation. Supplementation in infancy of preterm infants had no effect on IQ in later life. It may be due to the fact that infants born preterm miss out on the peak period of in utero DHA accretion to the brain during the last trimester of pregnancy (Kuipers et al. 2011), which is hypothesized to contribute to the increased prevalence of neurodevelopmental deficits in this population (Martinez 1992). These findings provide more complete and reliable evidence for the potential benefits of n–3 PUFA supplementation in cognitive, language, and intelligence development.

We found consistent conclusions were not reached for cognition with different scales. Based on the BSID‐II, the overall pooled results with n–3 supplementation.

For MDI showed significant improvement, however, based on the BSID‐III, indicated no significant improvement. MDI is used to assess cognitive and language development. Bayley‐III includes a separation of the original MDI into distinct cognitive, receptive language, and expressive language scales. Due to the correlation of Bayley‐III with the MDI scores appearing worse at lower test score values (Moore et al. 2012), we analysed the two outcomes separately. Furthermore, these scales were designed to detect developmental delays and may not be sensitive enough to detect subtle differences in neurodevelopment in healthy children (Dziechciarz, Horvath, and Szajewska 2010). Cognitive performance is partly influenced by genetics, nutrition, and a broad spectrum of environmental factors, especially the family environment for small children. In our included trials, a limited number of studies accounted for the family or home environment.

### 4.1 Prior Studies

Compared with the most recent meta‐analysis published in 2018 (Shulkin et al. 2018), our study added 11 studies, three on mothers and eight on infants (three on term infants, five on preterm infants). The periods of supplementation were divided more granularly. Supplementation for mothers included supplementation during pregnancy and/or lactation. To reduce the heterogeneity due to different measurement scales, we analysed BSID‐II and BSID‐III separately. This study is also the first meta‐analysis of n–3 PUFA supplementation based on BSID‐III. The current analysis yields similar conclusions partly. First, the overall pooled results for MDI showed significant improvement. Second, regarding PDI, combined results indicated no significant enhancement with n–3 supplementation.

However, the newly included studies utilizing the third version of BSID, potentially provided data for the cognitive and language composite analysis. The combined overall results or subgroup analyses for cognitive consistently indicated no significant improvement with supplementation. Only results from the subgroup of maternal supplementation showed a significant enhancement in language scores, while other subgroups revealed no notable improvement. The previous meta‐analysis (Shulkin et al. 2018), which combined BSID‐III (cognitive and language) and MDI of BSID‐II, suggested that supplementation with n–3 PUFA for preterm infants significantly benefited cognition. However, few studies of our analysis evaluated cognitive and language composite later in childhood, limiting power to assess this outcome.

We have updated results on whether fatty acid supplementation during pregnancy and lactation improves IQ in later childhood. In our review, pooled results of fatty acid supplementation in term infants showed significant improvements in IQ, as assessed by Wechsler series scales. Moreover, previous meta‐analyses showed no apparent difference in fatty acid supplementation (Shulkin et al. 2018), the scale used in addition to the Wechsler series scales and the K‐ABC/Stanford–Binet scales were also combined. Heterogeneity induced by different assessment scales is worth exploring. Although the correlations between the WASI‐II and the WISC‐IV were acceptable (*r* = 0.71) (McCrimmon and Smith 2013), the WPPSI and WISC share the Wechsler–Bellevue as a common ancestor and have many common characteristics. The changes may be in how the index scores and Full Scale Intelligence Quotient (FSIQ) are calculated (Willis, Dumont, and Kaufman 2015). In our findings, fatty acids supplementation in preterm infants had no significant effect on IQ, and two of the studies we included were at or near high dose supplementation (≈1% total fatty acids), consistent with the follow‐up results of the DINO randomized controlled trial at 7 years of age (Collins et al. 2015).

Furthermore, none of previous four systematic reviews showed a significant impact of adding PUFA to infant formula on cognitive development, although the cognitive development levels were mainly assessed using the BSID‐II (Beyerlein et al. 2010; Jasani et al. 2017; Moon et al. 2016; Qawasmi et al. 2012). Cochrane reviewed MDI comparison results on 12, 18, and 24 months, no significant differences were found (Moon et al. 2016).

### 4.2 Limitation

Potential limitations should be considered. First, some RCTs included not of high quality, only eight trials are in low risk in all seven domains based on the Cochrane quality assessment. More high‐quality studies are needed for combined analysis. Second, heterogeneity was high in some analysis, although we used random effects models and subgroup analyses. The DHA dose may be an essential factor to consider, particularly in preterm infants, who are at the most significant risk of DHA insufficiency. Due to insufficient data, it is impossible to analyse subgroups with different doses of DHA supplementation. Furthermore, the influence of genetic and environmental factors could not be assessed. In addition, the reliability of the results for the cognitive and language composite needs to be further improved by more studies based on BSID‐III.

## 5 Conclusion

Supplementation with n–3 PUFA during pregnancy or breastfeeding in mothers has increased language abilities. Furthermore, direct supplementation in term infants can improve intelligence in later childhood. However, insufficient evidence supports the claim that supplementation improves cognitive abilities. The effects of DHA supplementation on cognition still need to be discussed, or more trials are needed.

## Author Contributions

Yingyu Liu conducted the literature searches, articles selection, and analysis; interpreted the results; and drafted the article. Lijun Zhong reviewed the literature searches; interpreted the results; and drafted the article; He acted as a consultant when discrepancies were present in data extraction between reviewers. Yuan Feng and Qianlu Ding collaborated in the interpretation of the results and in drafting the article. Yujian Zhang did article selection, data extraction and participated in the interpretation of the results and reviewed the final version of the paper.

## Conflicts of Interest

The authors declare no conflicts of interest.

## Supporting information

Supporting information.

## Data Availability

The data that support the findings of this study are openly available in PubMed at https://pubmed.ncbi.nlm.nih.gov/29546296.
