# Supplementary material for: N–3 Fatty Acid Supplementation in Mothers and Infants for Childhood Psychomotor and Cognitive Development: An Updated Systematic Review and Meta‐Analysis
Source: Matern Child Nutr. 2024 Nov 28;21(2):e13767. doi: 10.1111/mcn.13767 (PMC11956035; doi:10.1111/mcn.13767)
Supplement: Supplementary file 1 — Supporting information. [file MCN-21-e13767-s001.docx]

**PART 1**

**Search strategy(Based on search strategies of previous meta-analysis:**Shulkin, M, Pimpin, L, Bellinger, D, et al. n-3 Fatty Acid Supplementation in Mothers, Preterm Infants, and Term Infants and Childhood Psychomotor and Visual Development: A Systematic Review and Meta-Analysis. J NUTR. 2018; 148 (3): 409-418. doi: 10.1093/jn/nxx031**)**

**PubMed search terms**

“fatty acids, omega-3”[MeSH] OR “fatty acid”[tiab] OR “fatty acids”[tiab] OR “fatty-acid”[tiab] OR “fatty-acids”[tiab] OR “omega-3”[tiab] OR “n-3”[tiab] OR “DHA”[tiab] OR “docosahexaenoic acid”[tiab] OR “EPA”[tiab] OR “eicosapentaenoic acid”[tiab] OR “essential fatty acid” [tiab] OR “essential fatty acids” [tiab] OR “LCPUFA”[tiab] OR “long chain polyunsaturated” [tiab] OR “long-chain polyunsaturated” [tiab] OR “PUFA”[tiab] OR “PUFAs”[tiab] OR “polyunsaturated fatty acid”[tiab] OR “polyunsaturated fatty acids”[tiab] OR “fish oils”[MeSH] OR “fish oil”[tiab] OR “fish proteins”[MeSH] OR “fishes”[MeSH] OR “fish products”[MeSH] OR “fish”[tiab] OR “seafood”[MeSH] OR “seafood”[tiab]

**AND**

neurodevelopment*[tiab] OR neurodevelopmental[tiab] OR “neural development"[tiab] OR “brain development”[tiab] OR “brain”[tiab] OR “neurological” [tiab] OR “cognitive development”[tiab] OR “cognition”[MeSH] OR “cognition”[tiab] OR “cognitive”[tiab] OR “mental development”[tiab] OR “psychomotor development” [tiab] OR “language”[MeSH] OR “language”[tiab] OR “intelligence”[tiab] OR “mental processes” [MeSH] OR “learning”[tiab] OR “executive function”[tiab] OR “memory”[tiab] OR “focus”[tiab] OR “attention”[tiab] OR “mental”[tiab] OR “visual acuity”[MeSH] OR “visual acuity”[tiab] OR “vision”[tiab] OR “eye development”[tiab] or “visual development”[tiab] or “visual function”[tiab] OR “retina”[tiab] OR “behavior”[MeSH] OR “behavior and behavior mechanisms”[MeSH] OR behavior*[tiab] OR behaviour*[tiab] OR “anxiety”[tiab] OR “emotions”[MeSH] OR emotion*[tiab] OR “emotional”[tiab] OR “mental health”[MeSH] OR “aggression”[tiab] OR “mood”[tiab] OR “psychosocial”[tiab] OR “depressive”[tiab] OR “developmental disabilities”[MeSH] OR “developmental disability”[tiab] OR “developmental delay”[tiab] OR “developmental delays”[tiab] OR “attention deficit”[tiab] OR “attention deficit and disruptive behavior disorders”[MeSH] OR “ADHD”[tiab] OR “autism”[tiab] OR "autistic"[tiab] OR “autistic disorder”[MeSH] OR “mental disorders”[MeSH] OR “intellectual disability”[tiab] OR “mental disorders diagnosed in childhood”[MeSH] OR “depression”[tiab] OR “bipolar”[tiab] OR “school”[tiab] OR “developmental score”[tiab] OR “developmental scores”[tiab] OR "IQ"[tiab] OR “intellectual quotient”[tiab] OR “neural index”[tiab] OR “neural indices"[tiab] OR “motor development”[tiab]

**AND**

“child”[MeSH] OR “infant”[MeSH] OR “pregnancy”[MeSH] OR “pregnant women” [MeSH] OR “child”[tiab] OR “children”[tiab] OR “childhood”[tiab] OR “infant”[tiab] OR “infants”[tiab] OR “pregnant”[tiab] OR “pregnancy”[tiab] OR “prenatal”[tiab] OR “maternal”[tiab] OR “lactation”[MeSH] OR “lactation”[tiab] OR “breast feeding”[MeSH] OR “breast-feed”[tiab] OR baby[tiab] OR babies[tiab]

**AND**

“clinical trial” [MeSH] OR “randomized controlled trial” [MeSH] OR “meta-analysis” [MeSH] OR “cohort studies” [MeSH] OR “longitudinal studies” [MeSH] OR “random allocation” [MeSH] OR “clinical trial” [ptyp] OR “controlled clinical trial” [ptyp] or “randomized controlled trial” [ptyp] OR “meta-analysis” [ptyp] OR “clinical trial” [tiab] OR “randomized controlled trial” [tiab] OR “trial” [tiab] OR “meta-analysis” [tiab] OR “cohort” [tiab] OR “longitudinal” [tiab] OR “random” [tiab] OR prospective[tiab] OR “comparative study”[ptyp] OR “multicenter study”[ptyp] OR risk assessment[MeSH]

**AND**

2016/01/01:2024/01/24[Date - Publication]

**Search strategy in other databases**

We adapted the PubMed search strategy for use in EMBASE, PsycInfo, and the Cochrane Library. Clinicaltrials.gov was searched on January 24, 2024 using the following search terms: “DHA” AND “development”

**PART 2**

| **Supplemental Table 1.** Study characteristics of maternal n-3 supplementation trials | | | | | | | | | | | | | | | | | |
| --- | --- | --- | --- | --- | --- | --- | --- | --- | --- | --- | --- | --- | --- | --- | --- | --- | --- |
| Author, year, trial | Country | Race | SES | Education | Maternal age, yr | Intervention period | Duration, wk | Compound given to both groups | Main intervention | Dose, mg/d | | | Control | Outcome | Latest age at outcome assessment, mo | | |
|  |  |  |  |  |  |  |  |  |  | DHA | EPA | AA |  |  | BSID | Visual^5^ | IQ |
| **Guillot2022[2]** | Canada | 56.8%white | 56.7%大于50000$ | high | 30.9 | within 72 hours of delivery until 36 weeks | 36 | - | DHA | 1200 | - | - | placebo | BSID-III | 18-22 |  |  |
| **Sass2021[3]** | Denmark | white | middle-class | .m | 32.3 | from the day of randomization until 1 week  after delivery |  |  | DHA+EPA | 37% | 55% |  | olive oil | BSID-III | 30 |  |  |
| **Colombo2019[1]** | Finland | 37%black | 45,599$ | high | 25.3 | 14.5  weeks of gestation until delivery | 14.5 | DSM Nutritional Products | DHA | 600 | - | - | soybean + corn oil | BSID/WPPSI | 18 | - | 72 |
| **Hurtado et al. 2015[6]** | Spain | White^1^ | .m | .m | 30.2 | 28th wk until delivery + 4 mo lactation | 29.2 | dairy drink with vitamins and minerals^2^ | DHA + EPA | 320 | 72 | - | Control dairy drink | BSID | 12 | - | - |
| **Meldrum 2015[4]** | Australia | White^1^ | .m | high | 31.8 | 20th wk until delivery | 20 | Vitamin E | DHA + EPA | 2200 | 1100 | - | Olive oil | WISC | - | - | 144 |
| **Ramakrishnan 2015[5]** | Mexico | Hispanic^1^ | med-low | average | 26.4 | 18-20th wk until delivery | 20 | - | DHA | 400 | 0 | - | Olive oil | BSID | 18 | - | - |
| **Makrides 2010, DOMInO[8]** | Australia | White^1^ | .m | average | 28.9 | 19th wk (median) until delivery | 21 | - | DHA + EPA | 800 | 100 | - | Vegetable oil | BSID-III | 18 | - | - |
| **Van Goor 2011[7]** | Netherlands | White^1^ | .m | average | 32.7 | 16.5th wk until delivery + 3 mo lactation | 36 | - | 1) DHA + EPA + higher AA | 220 | 36 | 220 | Soybean oil | BSID | 18 | - | - |
|  |  |  |  |  |  |  |  |  | 2) DHA + EPA + lower AA |  |  | 15 |  |  |  |  |  |
| **Jensen 2005[10]** | USA | 77% white | .m | high | 31.5 | Within 5 d of delivery, 4 mo lactation | 17.2 | - | DHA | 200 | 0 | - | Corn + soy oil | BSID | 30 | - | - |
| **Innis 2008[35]** | Canada | 73% white | med-high | high | 33.2 | 16th wk until delivery | 24 | - | DHA | 400 | 0 | - | Vegetable oil | TAC | - | 2 | - |
| **Judge 2007[36]** | USA | 93% black | low | average | 24.3 | 24th wk until delivery | 16 | cereal-based bar | DHA + EPA | 214 ^3^ | 26.80 ^4^ | - | Cereal bar with corn oil | TAC | - | 6 | - |
| **Tofail 2006[9]** | Bangladesh | Asian^1^ | low | low | 22.7 | 25th wk until delivery | 15 | - | DHA + EPA | 1200 | 1800 | - | Vegetable oil | BSID | 10 | - | - |
| **Lauritzen 2004[37]** | Denmark | White^1^ | .m | high | 29.9 | within 9 d of delivery, 4 mo lactation | 17.2 | - | DHA + EPA | 790 | 620 | 1.7% FA | Olive oil | VEP | - | 4 | - |
| **Gibson 1997[38]** | Australia | White^1^ | med | average | 30 | Within 5 d of delivery, 12 wk lactation | 12 | - | 1) DHA dose 1 | 200 | 0 | - | Oil, not further specified | VEP | - | 3.7 | - |
|  |  |  |  |  |  |  |  |  | 2) DHA dose 2 | 400 |  |  |  |  |  |  |  |
|  |  |  |  |  |  |  |  |  | 3) DHA dose 3 | 900 |  |  |  |  |  |  |  |
|  |  |  |  |  |  |  |  |  | 4) DHA dose 4 | 1300 |  |  |  |  |  |  |  |
| *Abbreviations: AA, arachidonic acid; BSID, Bayley Scales of Infant Development; DHA, docosahexaenoic acid; EPA, eicosapentaenoic acid; FA, fatty acids; IQ, Intelligence Quotient; .m, missing (not reported in publication); TAC, Teller Acuity Cards; VEP, Visual Evoked Potentials; WISC, Wechsler Intelligence Scale for Children.*  ^1^ Race not reported, assume based on predominant race in trial country, if country is racially homogenous.  ^2^ Vitamins and minerals included 80μg/100mL folic acid, 2.2 mg/100mL iron, 23 μg/100mL iodine, 0.4 μg/100mL vitamin B-12. Additionally, both groups received a recommended diet developed by a nutritionist, which highlighted the recommendation for weekly fish consumption.  ^3^ DHA dose from the following statement in methods section "women consumed 3, 5, or 7 bars weekly… the consumption of an average of 5 bars per week provided 214 mg DHA/day"  ^4^ EPA dose calculated from the 1:8 EPA:DHA ratio reported in the methods section.  ^5^ No new randomized controlled trials were added on the effects of n-3 PUFA supplementation on visual acuity(weighted mean difference), and the pooled results were consistent with previous meta-analyses(For maternal group: -0.02 (-0.04, 0.00))[51]. | | | | | | | | | | | | | | | | | |

| **Supplemental Table 2.** Study characteristics of term infant n-3 supplementation trials | | | | | | | | | | | | | | |
| --- | --- | --- | --- | --- | --- | --- | --- | --- | --- | --- | --- | --- | --- | --- |
| Author, year, trial | Country | Race | SES | Maternal education | Baseline age | Duration wk ^1^ | Main intervention | Dose, % FA^2^ | | | Outcome | Latest age at outcome assessment, mo | | |
|  |  |  |  |  |  |  |  | DHA | EPA | AA |  | BSID | Visual^11^ | IQ |
| **Suzanne 2020[14]** | Australia | white | .m | high | at birth | 24 | DHA+EPA | 250mg | 60mg | 0 | WASI-II | - | - | 72 |
| **Rebecca 2019[11]** | USA | 58% white, 36% black | .m | average | within 9 d of birth | 51.6 | DHA+AA | 0.96 | 0 | 0.64 | WISC-IV | - | - | 96-120 |
|  |  |  |  |  |  |  | DHA+AA | 0.64 | 0 | 0.64 |  |  |  |  |
|  |  |  |  |  |  |  | DHA+AA | 0.32 | 0 | 0.64 |  |  |  |  |
| **Devlin2017[13]** | Canada | 63.2%white | 62.7%大于$50000/year | High | 12month | 48 | DHA+AA | 200mg | 0 | 200mg | BSID-III | 24 | - | - |
| **Colombo 2013, DIAMOND *phase II Kansas City*[15]** | USA | 61% black | low | average | at birth | 51.6 | 1) 1:2 DHA:AA | 0.32 | 0 | 0.64 | BSID | 18 | - | - |
|  |  |  |  |  |  |  | 2) 1:1 DHA:AA | 0.64 |  |  |  |  |  |  |
|  |  |  |  |  |  |  | 3) 2:1 DHA:AA | 0.96 |  |  |  |  |  |  |
| **Willatts 2013[16]** | UK, Belgium, Italy | White^3^ | .m | average | within 3 d of birth | 17.2 | DHA + AA | 0.21 | 0 | 0.35 | WPPSI | - | - | 70 |
| **Meldrum 2012, IFOS[17]** | Australia | 91% white | high | high | at birth | 25.8 | DHA + EPA | 276 mg ^4^ | 100 mg | 0 | BSID-III | 18 | - | - |
| **Drover 2011[18] DIAMOND *phase II Dallas*** | USA | 69% white | .m | high | within 9 d of birth | 51.6 | 1) 1:2 DHA:AA | 0.32 | 0 | 0.64 | BSID | 18 | - | - |
|  |  | 69% white |  | high |  |  | 2) 1:1 DHA:AA | 0.64 | 0 | 0.64 | BSID | 18 | - | - |
|  |  | 69% white |  | high |  |  | 3) 2:1 DHA:AA | 0.96 | 0 | 0.64 | BSID | 18 | - | - |
| **Agostoni 2006[19]** | Western Europe | White^3^ | .m | .m | Mean 19 d after PKU diagnosis | 49.4 | DHA + AA | 0.3 | 0 | 0.7 | BSID | 12 | - | - |
| **Birch 2007[12]** | USA | 81% white | .m | high | within 4 d of birth; mean 2.1 d | 17 | 1) DHA | 0.35 | 0 | 0 | HOTV, WPPSI | - | 48 | 48 |
| **Birch 2000[48]** |  | 75% white |  |  |  |  |  |  |  |  | BSID | 18 | - | **-** |
| **Birch 2007** |  | 81% white |  |  |  |  | 2) DHA + AA | 0.36 | 0 | 0.72 | HOTV, WPPSI | - | 48 | 48 |
| **Birch 2000[48]** |  | 75% white |  |  |  |  |  |  |  |  | BSID | 18 | - | **-** |
| **Birch 2005[39]** | USA | 79% white | .m | high | within 5 d of birth; mean 3.6 d | 52 | DHA + AA | 0.36 | 0 | 0.72 | VEP | - | 12 | - |
| **Ben 2004[41]^10^** | China | Asian^3^ | .m | .m | within 7 d of birth | 25.8 | DHA + AA | 6.9 mg/L^5^ | 0 | 6.9 mg/L | BSID | 6 | - | - |
| **Hoffman 2004[40]** | USA | .m | .m | .m | after 6 mo breastfeeding | 25.8 | DHA + AA + EPA | 130  mg ^6^ | 4.5  mg | 88  mg | VEP | - | 12 | - |
| **Auestad 2003[42]** | USA | 84% white | .m | high | 2 d (median) after birth | 51.6 | 1) DHA + EPA | 0.23 | 0.07 | 0 | Stanford-Binet, TAC | - | 39 | 39 |
| **Scott 1998[49]** |  |  |  |  |  |  |  |  |  |  | BSID | 12 | - | - |
| **Auestad 2003[42]** |  |  |  |  |  |  | 2) DHA + AA | 0.12 | 0 | 0.43 | Stanford-Binet, TAC | - | 39 | 39 |
| **Scott 1998[49]** |  |  |  |  |  |  |  |  |  |  | BSID | 12 | - | - |
| **Hoffman 2003[43]** | USA | 93% white | .m | .m | after 4-6 mo breastfeeding | 30.1 | DHA + AA | 0.36 | 0 | 0.72 | VEP | - | 12 | - |
| **Birch 2002[20]** | USA | 77% white | .m | high | after 6 wk breastfeeding | 46 | DHA + AA | 0.36 | 0 | 0.72 | VEP | - | 12 | - |
| **Auestad 2001[21]** | USA | 85% white | .m | high | within 9 d of birth | 51.6 | 1) DHA + AA | 0.14 | 0 | 0.45 | BSID, TAC | 12 | 12 | **-** |
|  |  |  |  |  |  |  | 2) DHA + AA + EPA | 0.13 | ≤0.04 | 0.46 |  |  |  |  |
| **Makrides 2000[22]** | Australia | White^3^ | low | low | within 7 d of birth | 51.6 | 1) DHA + EPA | 0.35 | 0.1 | 0 | BSID, VEP | 24 | 7.9 | - |
|  |  |  |  |  |  |  | 2) DHA + AA | 0.34 | 0 | 0.34 |  |  |  |  |
| **Lucas 1999[23]** | UK | White^3^ | med | low | within 7 d of birth | 25.8 | DHA + AA + EPA | 0.32 | 0.01 | 0.3 | BSID, KPS ^7^ | 18 | - | - |
| **Jorgensen 1998[24]** | Denmark | White^3^ | .m | .m | 25 d to 4 mo old | 13.6 | DHA + EPA + AA^8^ | 0.32 | 0.38 | 0.06 | VEP | - | 4 | - |
| **Carlson 1996[44]** | USA | 90% black | low | .m | .m | 51.6 | DHA + AA | 0.1 | 0 | 0.43 | TAC | - | 12 | - |
| **Makrides 1995[45]** | Australia | White^3^ | low | low | within 4-6 d of birth | 30 | DHA + EPA + AA ^9^ | 0.36 | 0.58 | 0.01 | VEP | - | 7 | - |
| *Abbreviations: AA, arachidonic acid; BSID, Bayley Scales of Infant Development; DHA, docosahexaenoic acid; EPA, eicosapentaenoic acid; FA, fatty acids; IQ, Intelligence Quotient; KPS,*  *Knobloch, Passamanick, and Sherrads Developmental Screening Index; .m, missing (not reported in publication); TAC, Teller Acuity Cards; VEP, Visual Evoked Potentials; WPPSI, Wechsler Preschool and Primary Scale of Intelligence.* | | | | | | | | | | | | | | |
| ^1^ There was a range in supplementation start dates in within trials. Therefore, for trials starting supplementation within first 10 days after birth, we calculated a conservative supplementation duration using the full time period until supplementation end.  ^2^ Dose reported in % FA of infant formula, unless otherwise noted.  ^3^ Race not reported, assume based on predominant race in trial country, if country is racially homogenous.  ^4^ Infants in intervention group received a fish oil capsule; control infants received an image and scent-matched olive oil capsule.  ^5^ Intervention was infant formula, but dose was only reported in mg/L and could not be converted to %FA.  ^6^ Infants in intervention group received jarred baby food fortified with DHA-enriched egg yolk; control infants received the same baby food without the enriched egg yolk.  ^7^ Other infant development outcomes reporting overall developmental quotient, such as Brunet-Lezine and KPS, not included in meta-analysis.  ^8^ Trial included two intervention arms (+/- 0.5%FA of gamma-linolenic acid from borage oil), which were pooled in the outcome reported by the publication.  ^9^ Intervention included fish oil and evening primrose oil (which supplied gamma-linolenic acid) in a 1:1 ratio.  ^10^ Ben 2004[41] was not included in the meta-analysis because the bias assessment was 0, and analysis data were unavailable from the literature.  ^11^ No new randomized controlled trials were added on the effects of n-3 PUFA supplementation on visual acuity(weighted mean difference), and the pooled results were consistent with previous meta-analyses(For term infant group: -0.08 (-0.11, -0.05)[51]. | | | | | | | | | | | | | | |

| **Supplemental Table 3.** Study characteristics of pre-term infant n-3 supplementation trials | | | | | | | | | | | | | | |
| --- | --- | --- | --- | --- | --- | --- | --- | --- | --- | --- | --- | --- | --- | --- |
| Author, year | Country | Race | SES | Maternal education | GA, wk^1^ | Duration, wk ^2^ | Main intervention | Dose, %FA | | | Outcome | Latest age at outcome assessment, mo | | |
|  |  |  |  |  |  |  |  | DHA | EPA | AA |  | BSID | Visual^8^ | IQ |
| **Gould2022[28]** | Australia/ New Zealand /Singapore | White^3^ | .m | high | Less Than 29 Weeks | 36 weeks of post menstrual age | DHA | 61mg/kg/d |  |  | WPPSI | - | - | 60 |
| **Hewawasam 2021[26]** | Australia/ New Zealand /Singapore | White^3^ | .m | high | Less Than 29 Weeks | 36 weeks’ post menstrual age | DHA | 61mg/kg/d |  |  | BSID-III | 24 | - | - |
| **Boone2021[27]** | USA | 63% White | .m | .m | less than 35weeks | 24 | DHA+AA |  |  |  | BSID-III | 24 | - | - |
| **Keim 2018[25]** | USA | 60.3%white | .m | high | less than 35 weeks | 24 | DHA+AA | 200mg |  | 200mg | BSID-III | 15.7 | - | - |
| **Henriksen 2016[29]** | Norway | white | .m | average | gestational age of 28.6  (2.7) weeks | 9 | DHA+AA | 0.91 |  | 0.86 | WASI | - | - | 96 |
| **Isaacs 2011[30]** | UK | White^3^ | 23% high SES | low | 30.8 | 47.9 ^4^ | DHA + EPA + AA | 0.5 | 0.1 | 0.04 | WASI | - | - | 130 |
| **Fewtrell 2004[50]** |  |  |  |  | 31.2 | 47.5 |  |  |  |  | BSID, KPS ^5^ | 18 | - | - |
| **Fang 2005[31]** | Taiwan | Asian^3^ | .m | .m | 35.6 | 30.2 | DHA + AA | 0.05 | 0 | 0.1 | BSID, VEP | 12 | 6 | - |
| **Clandinin 2005[32]** | Australia | White^3^ | .m | .m | 29.4 | 60.9 | 1) DHA + AA | 0.32 ^6^ | 0 | 0.64 | BSID | 18 | - | - |
|  |  |  |  |  |  |  | 2) DHA + EPA + AA |  | 0.1 |  |  |  |  |  |
| **Van Wezel-Meijler 2002[33]** | Netherlands | White ^3^ | .m | .m | 30.4 | 35.4 | DHA + AA | 0.34 | 0 | 0.7 | BSID, TAC | 24 | 24 | - |
| **O’Connor 2001[34]** | USA, UK, Chile | 82% white | .m | .m | 29.8 | 61.9 | 1) DHA + EPA + AA | 0.18^7^ | 0.01 | 0.43 | BSID, VEP | 12 | 6 | - |
|  |  |  |  |  |  |  | 2) DHA + AA | 0.17 | 0 | 0.41 |  |  |  |  |
| **Uauy Dagach 1994[46]** | USA, Chile | .m | .m | .m | 30 | 27 | DHA + EPA + AA | 0.35 | 0.65 | 0.1 | VEP | - | 4 | - |
| **Carlson 1993[47]** | USA | 88% black | low | .m | 29 | 53.6 | DHA + EPA | 0.3 | 0.2 | 0 | TAC | - | 12 | - |
| *Abbreviations: AA, arachidonic acid; BSID, Bayley Scales of Infant Development; DHA, docosahexaenoic acid; EPA, eicosapentaenoic acid; FA, fatty acids; IQ, Intelligence Quotient; KPS,*  *Knobloch, Passamanick, and Sherrads Developmental Screening Index; .m, missing; TAC, Teller Acuity Cards; VEP, Visual Evoked Potentials; WASI, Wechsler Abbreviated Scale of Intelligence.*  ^1^ Gestational age at birth  ^2^ Supplementation duration for pre-term infants calculated taking into account duration until term gestational age. Supplementation start varied substantially (e.g. within 1-60 days of birth in one trial), but since mean start was within two weeks of birth for all trials except one, we calculated a conservative supplementation duration using gestational age at birth as follows when trials only reported supplementation end in months post-term: (40 weeks – mean gestational age at birth) + (supplementation end in months post-term * 4.3 weeks/month). In Carlson et al. 1993, where mean supplementation start was 25 days, we added 25 days to the calculation above.  ^3^ Race not reported, assume based on predominant race in trial country, if country is racially homogenous.  ^4^ Estimated duration is slightly different between Isaacs et al. 2011 and Fewtrell et al. 2004 because mean gestational age at birth was different.  ^5^ Other infant development outcomes, such as KPS, not included in meta-analysis.  ^6^ Two trials (one maternal, one pre-term infant) with two publications each were assigned different quality scores between publications. The analysis in Fewtrell et al. 2004 was performed on an intention-to-treat basis, which contributed to this publication being rated as having a low risk of bias of incomplete outcome assessment.  ^7^ When pre-term infant supplementation trials included formulas of varying fatty acid compositions before and after infants reached term corrected age (i.e. different pre- and post-term formulas), we calculated the weighted average dose of supplementation based on the reported doses and average duration between baseline and term corrected age (40 weeks minus average gestational age) and between term corrected age and intervention end. This calculated weighted average dose approximated the term formula composition since pre-term supplementation trials were at least 6 months in length.  ^8^ No new randomized controlled trials were added on the effects of n-3 PUFA supplementation on visual acuity(weighted mean difference), and the pooled results were consistent with previous meta-analyses(For pre-term infant group: -0.08 (-0.14, -0.01)[51]. | | | | | | | | | | | | | | |

**PART 3**

**Supplement Figure 1 Risk bias of included studies**.(+：low risk; -: high risk; ?: unclear risk)

(A)Risk bias of studies on maternal supplementation (B)Risk bias of studies on term infant supplementation (C)Risk bias of studies on preterm infant supplementation

**
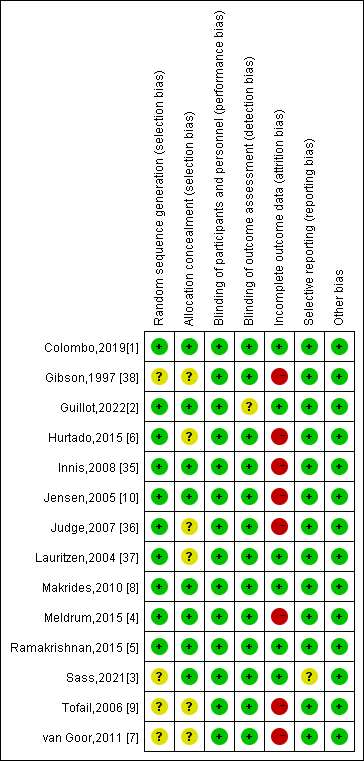

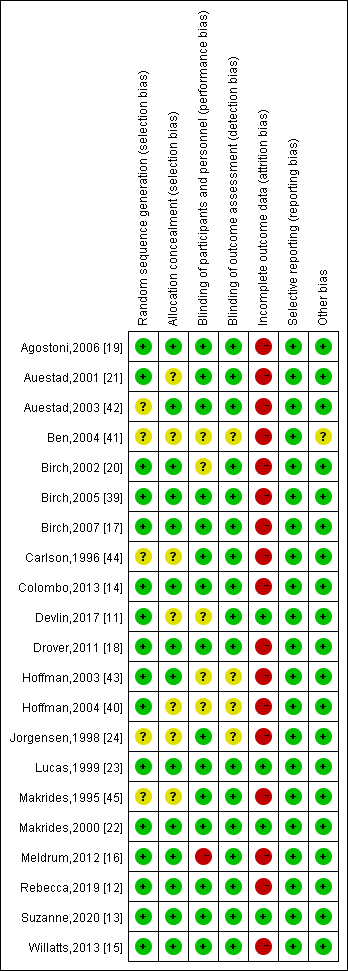

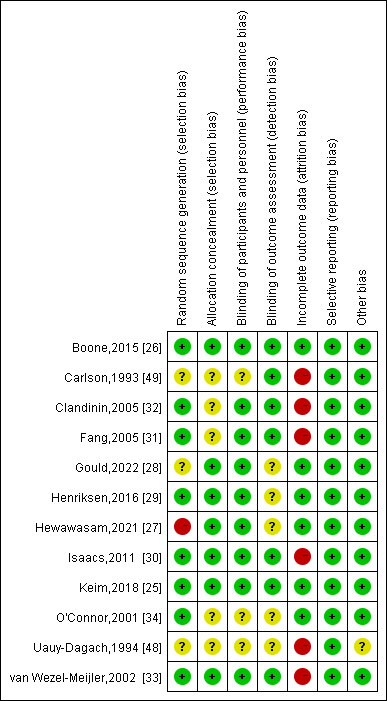
**

**A B C**

**Supplement Figure 2-A Forest of pooled results of Mental Development Index.** Effects of n-3 PUFA supplementation on MDI (weighted mean difference) in randomized controlled trials. These analyses included 30 intervention arms from 19 studies, with an overall pooled result across all supplementation periods of 1.91(95%CI:0.48-3.34) .Findings were pooled using random effects meta-analysis. Shaded squares represent the weight of each study, and dotted vertical lines and diamonds represent the pooled central estimate and its 95% CI, respectively, for each group. Age(mo):age at outcome assessment, months.


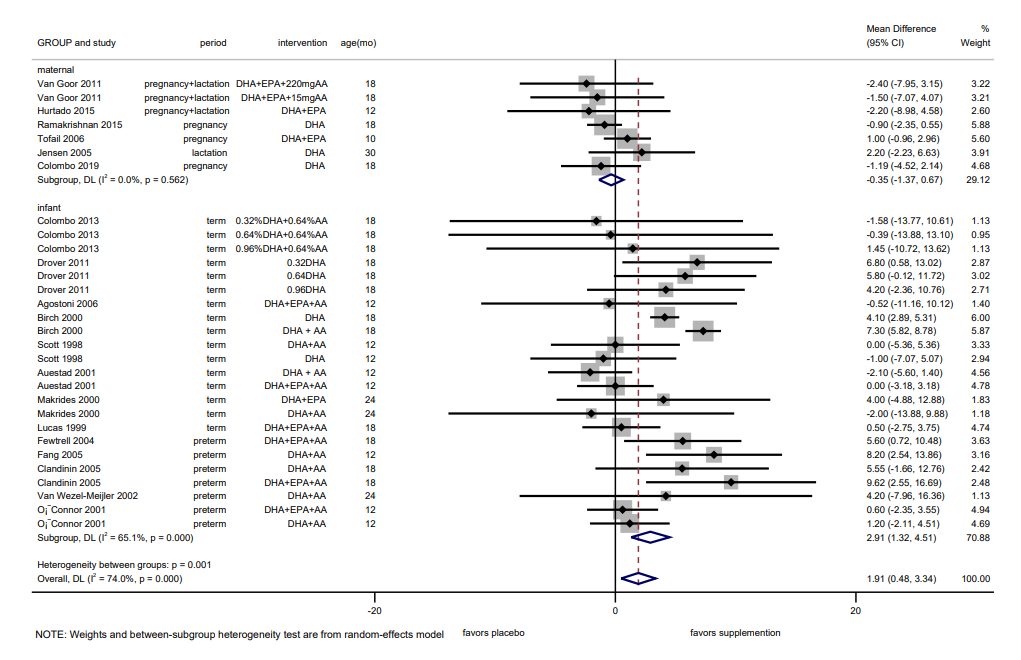


**Supplement Figure 2-B Forest of pooled results of Psychomotor Development Index.**Effects of n-3 PUFA supplementation on MDI (weighted mean difference) in randomized controlled trials. These analyses included 35 intervention arms from 24 studies, with an overall pooled result across all supplementation periods of 0.61(95%CI:-0.09-1.31) .Findings were pooled using random effects meta-analysis. Shaded squares represent the weight of each study, and dotted vertical lines and diamonds represent the pooled central estimate and its 95% CI, respectively, for each group.Age(mo):age at outcome assessment, months.


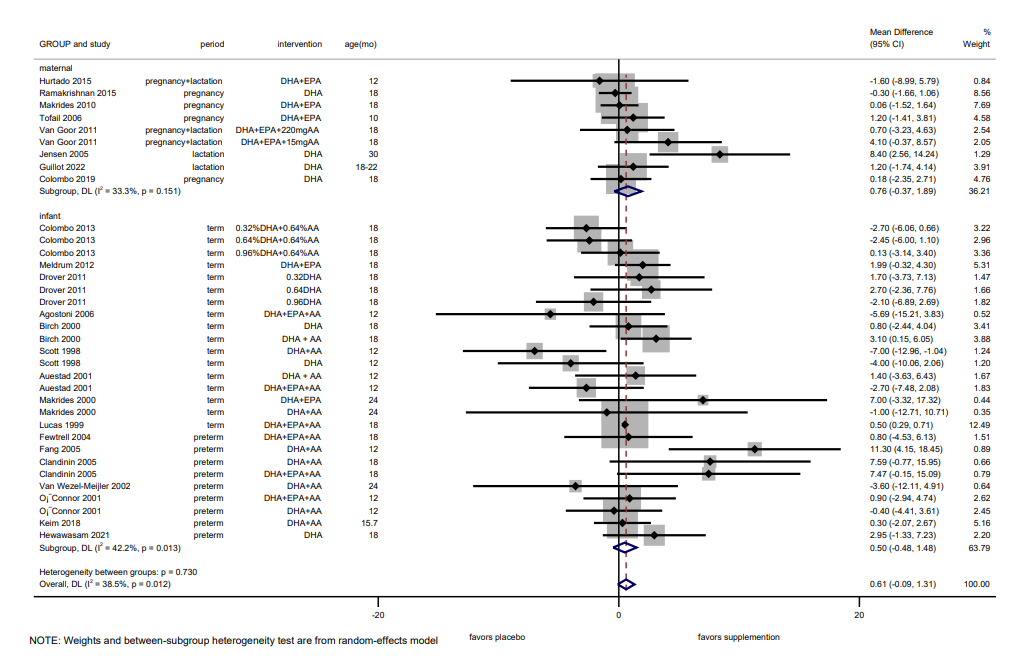


**Supplement Figure 2-C Forest of pooled results of cognitive composite.**Effects of n-3 PUFA supplementation on cognitive composite (weighted mean difference) in randomized controlled trials. These analyses included 8 intervention arms from 8 studies, with pooled result across all supplementation periods.Findings were pooled using random effects meta-analysis. Shaded squares represent the weight of each study, and dotted vertical lines and diamonds represent the pooled central estimate and its 95% CI, respectively, for each group.Age(mo):age at outcome assessment, months.


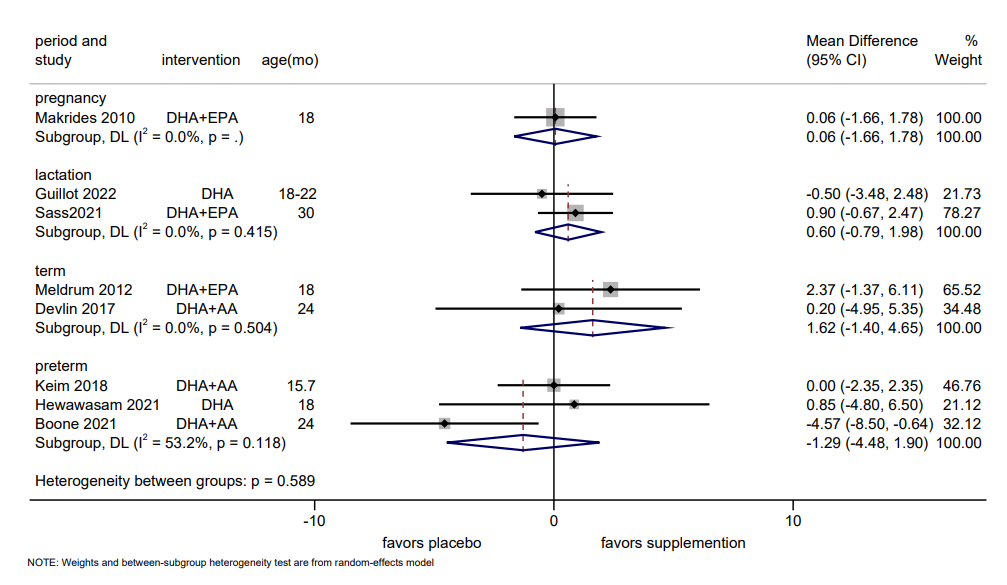


**Supplement Figure 2-D Forest of pooled results of language composite.**Effects of n-3 PUFA supplementation on language composite (weighted mean difference) in randomized controlled trials. These analyses included 7 intervention arms from 7 studies, with pooled result across all supplementation periods.Findings were pooled using random effects meta-analysis. Shaded squares represent the weight of each study, and dotted vertical lines and diamonds represent the pooled central estimate and its 95% CI, respectively, for each group.Age(mo):age at outcome assessment, months.


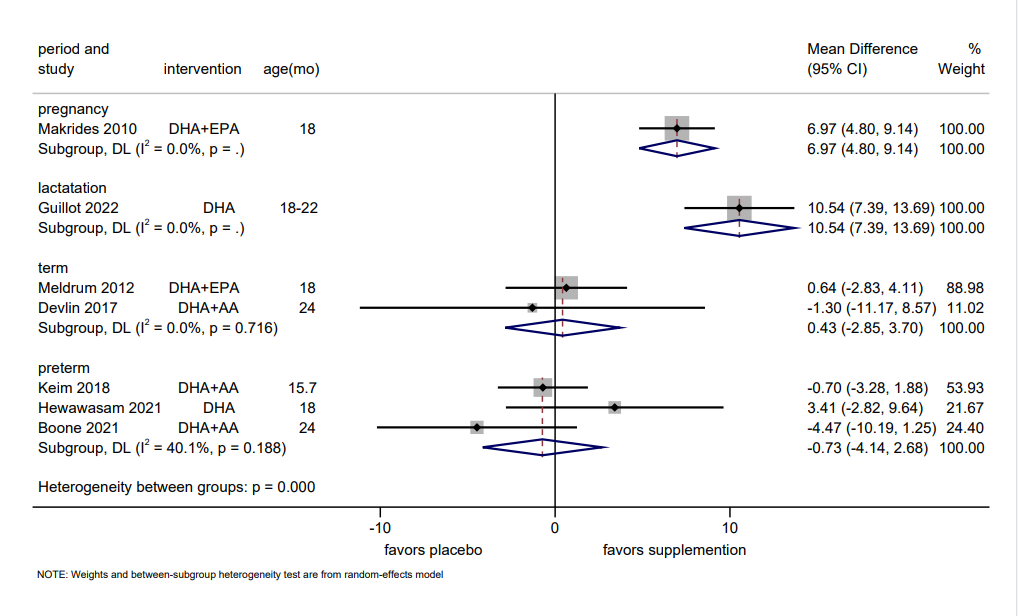


**Supplement Figure 2-E Forest of pooled results of IQ.**Effects of n-3 PUFA supplementation on IQ (weighted mean difference) in randomized controlled trials. These analyses included 13 intervention arms from 10 studies, with pooled result across all supplementation periods.Findings were pooled using random effects meta-analysis. Shaded squares represent the weight of each study, and dotted vertical lines and diamonds represent the pooled central estimate and its 95% CI, respectively, for each group.Age(mo):age at outcome assessment, months.


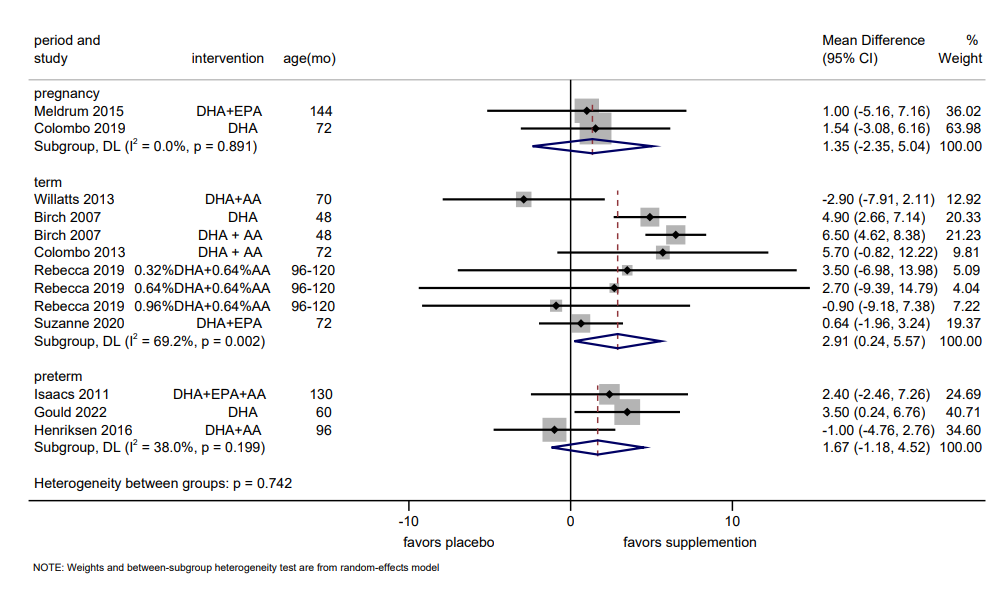


**Supplement Figure 3 Funnel plots graph variance (SE) versus effect size (MD), to assess asymmetry. A)** Bayley Scales of Infant Development (BSID-II)- Mental Developmental Index (MDI) (*n*=30 intervention arms); Egger’s Test P=0.389. **B**) BSID-II-Psychomotor Developmental Index (PDI) (*n=*35 intervention arms); Egger’s Test P=0.595. **C)** Bayley Scales of Infant Development (BSID-III)-Cognitive composite(*n*=8 intervention arms); Egger’s Test P=0.525. **D)** BSID-III-Language composite(*n*=7 intervention arm); Egger’s Test P=0.432**. E)** Intelligence Quotient (*n=*13 intervention arms); Egger’s Test P=0.091.


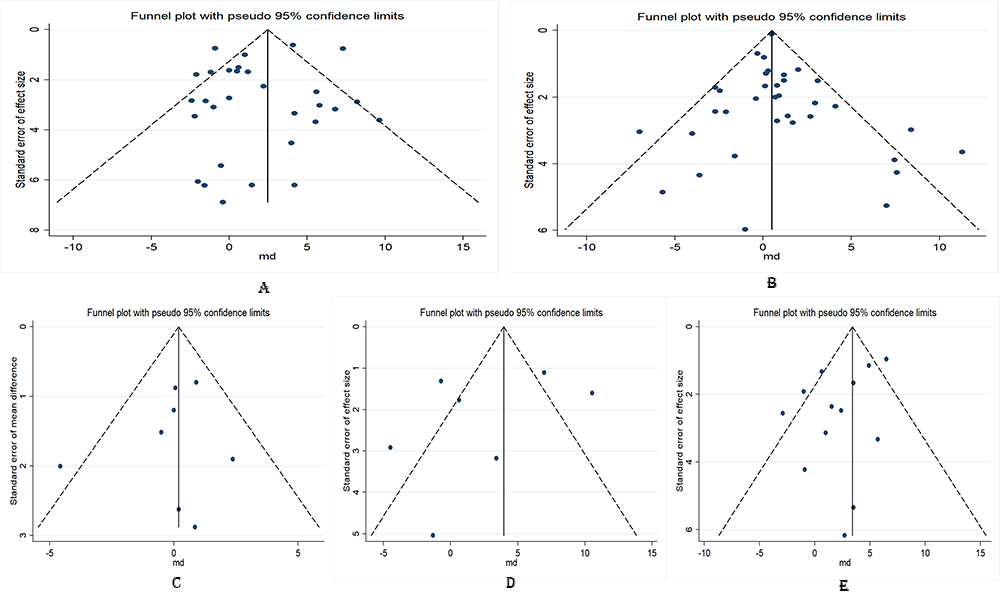


***Reference***

1. Colombo J, Jill Shaddy D, Gustafson K, et al. The Kansas University DHA Outcomes Study (KUDOS) clinical trial: Long-term behavioral follow-up of the effects of prenatal DHA supplementation. American Journal of Clinical Nutrition. 2019;109(5):1380-1392.

2. Guillot M, Synnes A, Pronovost E, et al. Maternal High-Dose DHA Supplementation and Neurodevelopment at 18-22 Months of Preterm Children. Pediatrics. 2022;150(1).

3. Sass L, Bjarnadóttir E, Stokholm J, et al. Fish oil supplementation in pregnancy and neurodevelopment in childhood— A randomized clinical trial. Child Development. 2021;92(4):1624-1635.

4. Meldrum S, Dunstan JA, Foster JK, Simmer K, Prescott SL. Maternal fish oil supplementation in pregnancy: a 12 year follow-up of a randomised controlled trial. Nutrients. 2015;7(3).

5. Ramakrishnan U, Stinger A, DiGirolamo AM, et al. Prenatal Docosahexaenoic Acid Supplementation and Offspring Development at 18 Months: Randomized Controlled Trial. PloS one. 2015;10(8):e0120065.

6. Hurtado JA, Iznaola C Fau - Peña M, Peña M Fau - Ruíz J, et al. Effects of Maternal Ω-3 Supplementation on Fatty Acids and on Visual and Cognitive Development. JOURNAL OF PEDIATRIC GASTROENTEROLOGY AND NUTRITION. 2015;61(4):472-480.

7. van Goor SA, Dijck-Brouwer Da Fau - Erwich JJHM, Erwich Jj Fau - Schaafsma A, Schaafsma A Fau - Hadders-Algra M, Hadders-Algra M. The influence of supplemental docosahexaenoic and arachidonic acids during pregnancy and lactation on neurodevelopment at eighteen months. PROSTAGLANDINS LEUKOTRIENES AND ESSENTIAL FATTY ACIDS. 2011;84(5-6):139-146.

8. Makrides M, Gibson Ra Fau - McPhee AJ, McPhee Aj Fau - Yelland L, Yelland L Fau - Quinlivan J, Quinlivan J Fau - Ryan P, Ryan P. Effect of DHA supplementation during pregnancy on maternal depression and neurodevelopment of young children: a randomized controlled trial. OBSTETRICAL & GYNECOLOGICAL SURVEY. 2011;66(2):79-81.

9. Tofail F, Kabir I Fau - Hamadani JD, Hamadani Jd Fau - Chowdhury F, et al. Supplementation of fish-oil and soy-oil during pregnancy and psychomotor development of infants. JOURNAL OF HEALTH POPULATION AND NUTRITION. 2006;24(1):48-56.

10. Jensen CL, Voigt Rg Fau - Prager TC, Prager Tc Fau - Zou YL, et al. Effects of maternal docosahexaenoic acid intake on visual function and neurodevelopment in breastfed term infants. AMERICAN JOURNAL OF CLINICAL NUTRITION. 2005;82(1):125-132.

11. Lepping RJ, Honea RA, Martin LE, et al. Long-chain polyunsaturated fatty acid supplementation in the first year of life affects brain function, structure, and metabolism at age nine years. Developmental psychobiology. 2019;61(1):5‐16.

12. Birch EE, Garfield S Fau - Castañeda Y, Castañeda Y Fau - Hughbanks-Wheaton D, Hughbanks-Wheaton D Fau - Uauy R, Uauy R Fau - Hoffman D, Hoffman D. Visual acuity and cognitive outcomes at 4 years of age in a double-blind, randomized trial of long-chain polyunsaturated fatty acid-supplemented infant formula. EARLY HUMAN DEVELOPMENT. 2007;83(5):279-284.

13. Devlin AM, Chau CMY, Dyer R, et al. Developmental Outcomes at 24 Months of Age in Toddlers Supplemented with Arachidonic Acid and Docosahexaenoic Acid: results of a Double Blind Randomized, Controlled Trial. Nutrients. 2017;9(9).

14. Meldrum SJ, Heaton AE, Foster JK, Prescott SL, Simmer Ao K. Do infants of breast-feeding mothers benefit from additional LCPUFA from fish oil? A six-year follow up. British journal of nutrition. 2020.

15. Colombo J, Carlson Se Fau - Cheatham CL, Cheatham Cl Fau - Shaddy DJ, et al. Long-term effects of LCPUFA supplementation on childhood cognitive outcomes. AMERICAN JOURNAL OF CLINICAL NUTRITION. 2013;98(2):403-412.

16. Willatts P, Forsyth S Fau - Agostoni C, Agostoni C Fau - Casaer P, Casaer P Fau - Riva E, Riva E Fau - Boehm G, Boehm G. Effects of long-chain PUFA supplementation in infant formula on cognitive function in later childhood. AMERICAN JOURNAL OF CLINICAL NUTRITION. 2013;98(2):536S-542S.

17. Meldrum SJ, D'Vaz N Fau - Simmer K, Simmer K Fau - Dunstan JA, Dunstan Ja Fau - Hird K, Hird K Fau - Prescott SL, Prescott SL. Effects of high-dose fish oil supplementation during early infancy on neurodevelopment and language: a randomised controlled trial. BRITISH JOURNAL OF NUTRITION. 2012;108(8):1443-1454.

18. Drover JR, Hoffman Dr Fau - Castañeda YS, Castañeda Ys Fau - Morale SE, et al. Cognitive function in 18-month-old term infants of the DIAMOND study: a randomized, controlled clinical trial with multiple dietary levels of docosahexaenoic acid. EARLY HUMAN DEVELOPMENT. 2011;87(3):223-230.

19. Agostoni C, Harvie A Fau - McCulloch DL, McCulloch Dl Fau - Demellweek C, et al. A randomized trial of long-chain polyunsaturated fatty acid supplementation in infants with phenylketonuria. DEVELOPMENTAL MEDICINE AND CHILD NEUROLOGY. 2006;48(3):207-212.

20. Birch EE, Hoffman Dr Fau - Castañeda YS, Castañeda Ys Fau - Fawcett SL, Fawcett Sl Fau - Birch DG, Birch Dg Fau - Uauy RD, Uauy RD. A randomized controlled trial of long-chain polyunsaturated fatty acid supplementation of formula in term infants after weaning at 6 wk of age. AMERICAN JOURNAL OF CLINICAL NUTRITION. 2002;75(3):570-580.

21. Auestad N, Halter R Fau - Hall RT, Hall Rt Fau - Blatter M, et al. Growth and development in term infants fed long-chain polyunsaturated fatty acids: a double-masked, randomized, parallel, prospective, multivariate study. PEDIATRICS. 2001;108(2):372-381.

22. Makrides M, Neumann Ma Fau - Simmer K, Simmer K Fau - Gibson RA, Gibson RA. A critical appraisal of the role of dietary long-chain polyunsaturated fatty acids on neural indices of term infants: a randomized, controlled trial. PEDIATRICS. 2000;105(1):32-38.

23. Lucas A, Stafford M Fau - Morley R, Morley R Fau - Abbott R, et al. Efficacy and safety of long-chain polyunsaturated fatty acid supplementation of infant-formula milk: a randomised trial. LANCET. 1999;354(9194):1948-1954.

24. Hørby Jørgensen M, Hølmer G Fau - Lund P, Lund P Fau - Hernell O, Hernell O Fau - Michaelsen KF, Michaelsen KF. Effect of formula supplemented with docosahexaenoic acid and gamma-linolenic acid on fatty acid status and visual acuity in term infants. JOURNAL OF PEDIATRIC GASTROENTEROLOGY AND NUTRITION. 1998;26(4):412-421.

25. Keim SA, Boone KM, Klebanoff MA, et al. Effect of Docosahexaenoic Acid Supplementation vs Placebo on Developmental Outcomes of Toddlers Born Preterm: a Randomized Clinical Trial. JAMA pediatrics. 2018;172(12):1126‐1134.

26. Hewawasam E, Collins CT, Muhlhausler BS, et al. DHA supplementation in infants born preterm and the effect on attention at 18 months' corrected age: follow-up of a subset of the N3RO randomised controlled trial. British journal of nutrition. 2021;125(4):420‐431.

27. Boone KM, Pattison K, Pelak G, et al. Docosahexaenoic and arachidonic acid supplementation at 1 year has mixed effects on development and behaviour at age 2 for preterm children. Acta Paediatrica. 2021;110(7):2082-2083.

28. Gould JF, Makrides M, Gibson RA, et al. Neonatal Docosahexaenoic Acid in Preterm Infants and Intelligence at 5 Years. New England journal of medicine. 2022;387(17):1579‐1588.

29. Henriksen C, Almaas AN, Westerberg AC, Drevon CA, Iversen PO, Nakstad B. Growth, metabolic markers, and cognition in 8-year old children born prematurely, follow-up of a randomized controlled trial with essential fatty acids. European journal of pediatrics. 2016;175(9):1165‐1174.

30. Isaacs EB, Ross S Fau - Kennedy K, Kennedy K Fau - Weaver LT, Weaver Lt Fau - Lucas A, Lucas A Fau - Fewtrell MS, Fewtrell MS. 10-year cognition in preterms after random assignment to fatty acid supplementation in infancy. PEDIATRICS. 2011;128(4):e890-898.

31. Fang PC, Kuo Hk Fau - Huang C-B, Huang Cb Fau - Ko T-Y, Ko Ty Fau - Chen C-C, Chen Cc Fau - Chung M-Y, Chung MY. The effect of supplementation of docosahexaenoic acid and arachidonic acid on visual acuity and neurodevelopment in larger preterm infants. Chang Gung medical journal. 2005;28(10):708-715.

32. Clandinin MT, Van Aerde Je Fau - Merkel KL, Merkel Kl Fau - Harris CL, et al. Growth and development of preterm infants fed infant formulas containing docosahexaenoic acid and arachidonic acid. JOURNAL OF PEDIATRICS. 2005;146(4):461-468.

33. van Wezel-Meijler G, van der Knaap Ms Fau - Huisman J, Huisman J Fau - Jonkman EJ, Jonkman Ej Fau - Valk J, Valk J Fau - Lafeber HN, Lafeber HN. Dietary supplementation of long-chain polyunsaturated fatty acids in preterm infants: effects on cerebral maturation. ACTA PAEDIATRICA. 2002;91(9):942-950.

34. O'Connor DL, Hall R Fau - Adamkin D, Adamkin D Fau - Auestad N, et al. Growth and development in preterm infants fed long-chain polyunsaturated fatty acids: a prospective, randomized controlled trial. PEDIATRICS. 2001;108(2):359-371

35. Innis SM, Friesen RW. Essential n-3 fatty acids in pregnant women and early visual acuity maturation in term infants. Am J Clin Nutr 2008;87(3):548-57.

36. Judge MP, Harel O, Lammi-Keefe CJ. A docosahexaenoic acid-functional food during pregnancy benefits infant visual acuity at four but not six months of age. Lipids 2007;42(2):117-22. doi: 10.1007/s11745-006-3007-3.

37. Lauritzen L, Jorgensen MH, Mikkelsen TB, Skovgaard l M, Straarup EM, Olsen SF, Hoy CE, Michaelsen KF. Maternal fish oil supplementation in lactation: effect on visual acuity and n-3 fatty acid content of infant erythrocytes. Lipids 2004;39(3):195-206.

38. Gibson RA, Neumann MA, Makrides M. Effect of increasing breast milk docosahexaenoic acid on plasma and erythrocyte phospholipid fatty acids and neural indices of exclusively breast fed infants. Eur J Clin Nutr 1997;51(9):578-84.

39. Birch EE, Castaneda YS, Wheaton DH, Birch DG, Uauy RD, Hoffman DR. Visual maturation of term infants fed long-chain polyunsaturated fatty acid-supplemented or control formula for 12 mo. Am J Clin Nutr 2005;81(4):871-9.

40. Hoffman DR, Theuer RC, Castaneda YS, Wheaton DH, Bosworth RG, O'Connor AR, Morale SE, Wiedemann LE, Birch EE. Maturation of visual acuity is accelerated in breast-fed term infants fed baby food containing DHA-enriched egg yolk. J Nutr 2004;134(9):2307-13.

41. Ben XM, Zhou XY, Zhao WH, Yu WL, Pan W, Zhang WL, Wu SM, Van Beusekom CM, Schaafsma A. Growth and development of term infants fed with milk with long-chain polyunsaturated fatty acid supplementation. Chin Med J (Engl) 2004;117(8):1268-70.

42. Auestad N, Scott DT, Janowsky JS, Jacobsen C, Carroll RE, Montalto MB, Halter R, Qiu W, Jacobs JR, Connor WE, et al. Visual, cognitive, and language assessments at 39 months: a follow-up study of children fed formulas containing long-chain polyunsaturated fatty acids to 1 year of age. Pediatrics 2003;112(3 Pt 1):e177-83.

43. Hoffman DR, Birch EE, Castaneda YS, Fawcett SL, Wheaton DH, Birch DG, Uauy R. Visual function in breast-fed term infants weaned to formula with or without long-chain polyunsaturates at 4 to 6 months: a randomized clinical trial. The Journal of pediatrics 2003;142(6):669-77. doi: 10.1067/mpd.2003.213.

44. Carlson SE, Ford AJ, Werkman SH, Peeples JM, Koo WW. Visual acuity and fatty acid status of term infants fed human milk and formulas with and without docosahexaenoate and arachidonate from egg yolk lecithin. Pediatric research 1996;39(5):882-8. doi: 10.1203/00006450-199605000-00024.

45. Makrides M, Neumann M, Simmer K, Pater J, Gibson R. Are long-chain polyunsaturated fatty acids essential nutrients in infancy? Lancet 1995;345(8963):1463-8.

46. Uauy-Dagach R, Mena P, Hoffman DR. Essential fatty acid metabolism and requirements for LBW infants. Acta paediatrica (Oslo, Norway : 1992) Supplement 1994;405:78-85.

47. Carlson SE, Werkman SH, Rhodes PG, Tolley EA. Visual-acuity development in healthy preterm infants: effect of marine-oil supplementation. Am J Clin Nutr 1993;58(1):35-42.

48. Birch EE, Garfield S, Hoffman DR, Uauy R, Birch DG. A randomized controlled trial of early dietary supply of long-chain polyunsaturated fatty acids and mental development in term infants. Dev Med Child Neurol 2000;42(3):174-81

49. Scott DT, Janowsky JS, Carroll RE, Taylor JA, Auestad N, Montalto MB. Formula supplementation with long-chain polyunsaturated fatty acids: are there developmental benefits? Pediatrics 1998;102(5):E59.

50. Fewtrell MS, Abbott RA, Kennedy K, Singhal A, Morley R, Caine E, Jamieson C, Cockburn F, Lucas A. Randomized, double-blind trial of long-chain polyunsaturated fatty acid supplementation with fish oil and borage oil in preterm infants. The Journal of pediatrics 2004;144(4):471-9. doi: 10.1016/j.jpeds.2004.01.034.

51. Shulkin, M., Pimpin, L., Bellinger, D., Kranz, S., Fawzi, W., Duggan, C., & Mozaffarian, D. (2018). n-3 Fatty Acid Supplementation in Mothers, Preterm Infants, and Term Infants and Childhood Psychomotor and Visual Development: A Systematic Review and Meta-Analysis. J Nutr, 148(3), 409-418. doi:10.1093/jn/nxx031
